# Secondhand Tobacco Exposure Assessed Using Urinary Cotinine Among 10-Year-Old Children in Japan: An 11-Year Repeated Cross-sectional Study

**DOI:** 10.1093/ntr/ntae220

**Published:** 2024-09-19

**Authors:** Yudai Tamada, Kenji Takeuchi, Takahiro Tabuchi

**Affiliations:** Department of International and Community Oral Health, Tohoku University Graduate School of Dentistry, Miyagi, Japan; Department of International and Community Oral Health, Tohoku University Graduate School of Dentistry, Miyagi, Japan; Division of Statistics and Data Science, Liaison Center for Innovative Dentistry, Tohoku University Graduate School of Dentistry, Miyagi, Japan; Division of Epidemiology, School of Public Health, Tohoku University Graduate School of Medicine, Miyagi, Japan

## Abstract

**Introduction:**

The emergence of heated tobacco products (HTPs) has made it important to monitor HTP-generated aerosols in addition to combustible cigarette (CC) smoke as a source of secondhand tobacco (SHT) exposure. We investigated the trend of SHT exposure in school-aged children and assessed whether SHT exposure depended on household tobacco use status.

**Aims and Methods:**

This repeated cross-sectional study from 2011 to 2021 (15 927 participants) was based on data from an annual survey of fourth-grade students (aged 10 years) in Kumagaya City, Japan. In addition to a questionnaire which includes questions about household tobacco use status, we measured the urinary cotinine levels of each participant by their first-morning urine sample to objectively assess SHT exposure. We defined the participants with urinary cotinine levels ≥5.0 ng/mL as being exposed to SHT.

**Results:**

The prevalence of SHT exposure decreased over the 11-year period from 18.6% in 2011 to 5.3% in 2021. It was significantly higher in households with tobacco users than without tobacco users (*t*-test *p*< .001). Prevalence of SHT exposure was 1.4% among the 68.1% of households not using tobacco, 22.9% among the 16.5% using only CC, 3.1% among the 12.3% using only HTP, and 27.6% among the 3.9% of households using CC and HTP.

**Conclusions:**

While the prevalence of SHT exposure showed a decreasing trend from 2011 to 2021, the prevalence of SHT exposure was higher in children with household members using tobacco products, regardless of the type of tobacco product, than in those without tobacco users.

**Implications:**

This study observed that the prevalence of SHT exposure was higher among children in households with tobacco users than among those without tobacco users, regardless of the type of tobacco product. Our findings highlight the importance of advocating that HTPs do not reduce the likelihood of SHT exposure to bystanders.

## Introduction

Accumulating evidence suggests that secondhand smoke has detrimental effects on the health of nonsmokers, including premature death.^1^ Secondhand smoke is one of the most important public health problems during childhood, with children accounting for 28% of deaths from secondhand smoke.^2^ Children are particularly vulnerable to secondhand smoke because of their high breathing rate and large lung surface area.^3^ Middle ear disease, asthma, wheezing, coughing, bronchitis, pneumonia, and pulmonary dysfunction are common in children exposed to secondhand smoke.^4,5^ Recently, secondhand smoke exposure in childhood is reported to be associated with an increased risk of death due to respiratory disease, coronary heart disease, and pancreatic cancer in adulthood.^6–8^

Japan has been a major market for heated tobacco products (HTPs) since November 2014, when IQOS, one of the most popular brands of HTPs, was launched before other countries. The number of HTP users has increased rapidly in recent years, with younger people in the child-rearing generation being more likely to use HTPs.^9^ HTPs are the second-most used tobacco product after traditional combustible cigarettes (CCs) in Japan, with the percentage users of CCs and HTPs estimated to be 19.4% and 11.8%, respectively, in 2022.^10^ Against the background of the rapid spread of HTPs use, our recent study estimated that approximately one in 10 people in Japan were already exposed to secondhand aerosols from HTPs, as of 2020.^11^

The emergence of HTPs has complicated the issue of secondhand tobacco (SHT) exposure, such as secondhand exposure to CC smoke and HTP-generated aerosols. Although aerosols generated from HTPs contain significantly lower amounts of harmful and potentially harmful constituents than those generated in CC smoke,^12–15^ there is currently no evidence to indicate that HTPs are less harmful than conventional tobacco products.^16,17^ A recent study reported that secondhand aerosol exposure from HTPs was associated with asthma attacks/asthma-like symptoms and persistent cough among current non-tobacco users,^18^ suggesting that both HTP-generated aerosols and CC smoke should be treated as a public health problem of SHT exposure. In other words, it is important to monitor aerosol exposure from HTPs, not only for HTP users but also for bystanders, including children.

Although SHT exposure assessed based on self-reporting may be subject to response bias,^19^ biomonitoring of the urinary levels of nicotine metabolites, such as cotinine, is a more accurate and widely used method to assess SHT exposure (people who were exposed to SHT, regardless of tobacco type, has a higher level of urinary cotinine).^20–22^ Previous studies, which assessed SHT exposure by urinary cotinine levels, investigated the trends in the prevalence of SHT exposure in nonsmoking adults^23,24^; however, to the best of our knowledge, there have been no such studies in school-aged children, especially elementary school children.^25–27^ Therefore, the main purposes of this study were to evaluate the trends in the prevalence of SHT exposure, assessed by urinary cotinine levels, among elementary school children in Japan from 2011 to 2021 and to examine the association between household tobacco product use and the likelihood of being exposed to SHT.

## Methods

### Study Population and Setting

This repeated cross-sectional study was based on data from an annual survey conducted in Kumagaya City from 2011 to 2021 by the Education Board to investigate the prevalence of SHT exposure among school-aged children. Kumagaya City is in the Saitama Prefecture, Kanto Region, Japan, and has a residential population of approximately 200 000 people. All fourth-grade children (aged 10 years) at elementary schools in Kumagaya City were recruited to participate in the survey. The urinary cotinine level of each child was measured to objectively assess whether they were exposed to SHT. In addition, parents or guardians were asked to answer a questionnaire that included questions on the tobacco use status of household members. From the 17 596 children with information from the questionnaire results, we extracted 15 927 children with information on a urinary cotinine level, as the analytical sample. The number of participants who did not have information on the urinary cotinine level, by survey year, from 2011 to 2021 is shown in Table S1.

This study included two analyses (analyses 1 and 2) with different purposes. Analysis 1 investigated the annual trend in the prevalence of SHT exposure, assessed by urinary cotinine levels. Analysis 2 assessed the prevalence of SHT exposure, assessed by urinary cotinine levels, according to household tobacco use status, that is, a stratified analysis by product type. While analysis 1 was based on data from 2011 to 2021, analysis 2 used data from 2018 to 2021 because the question regarding HTP use status was included in the questionnaire from 2018.

### Urinary Cotinine Measurement and Definition of SHT Exposure

Children were instructed to collect their first morning urine sample in a sterile container, provided in advance. The samples were sent from their schools to a designated laboratory (Cosmic Corporation Co., Ltd., Tokyo, Japan; BML Inc., Tokyo, Japan) on the same day. The urinary cotinine level was measured using a monoclonal antibody-based competitive enzyme-linked immunosorbent assay. Using this method, the limit of quantitation for urinary cotinine levels was 1.3 ng/mL. According to the definition by the Japan Society for Tobacco Control^28^ and the definitions used in a previous study,^29^ we classified the children with a urinary cotinine level ≥5.0 ng/mL into the cotinine-assessed SHT exposed group. We used this definition partly because our method could not differentiate the source of SHT, that is, CC smoke or HTP-generated aerosols, although there might be differences in containing cotinine levels. In the sensitivity analysis, given that various cutoff was used to define SHT exposure in previous studies^19,30^ and using a single cutoff may not capture the full picture of the current situation, we repeated the analyses using a different cutoff (≥3.0 ng/mL) in the definition of SHT exposure.

### Household Tobacco Use

Household tobacco use status was defined as the presence of at least one household member who used tobacco products at home, based on the responses of family members, specifically fathers, mothers, and other cohabitants, to the following standard single-item questions: “Do you use tobacco products?” If the answer was “daily” or “sometimes,” the respondent was treated as a tobacco user; if the answer was “former” or “never,” the respondent was treated as a non-tobacco user. For tobacco product-specific use, household members were asked about the number of each of the CCs and HTPs used at home on the day before the children’s urine samples were collected, based on the following question: “What type and number of tobacco products did you use the day before your child’s urine sample was collected?” Following the definitions in previous studies,^10,31,32^ we treated respondents as “CC users” if they used one or more CCs at home and “HTP users” if they used one or more HTPs at home, that is, respondents who used at least one CC or HTP regarded as current tobacco users. Subsequently, we defined household tobacco use status by aggregating the household members’ tobacco product use status at the household level into the following four categories: none, only CCs, only HTPs, and CCs and HTPs. Since the sale of nicotine-containing e-cigarettes is prohibited in Japan, the questionnaire did not include a question on e-cigarette use.

### Statistical Analysis

In analysis 1, we constructed a scatter plot of urinary cotinine levels and a forest plot of the prevalence of SHT exposure by survey year from 2011 to 2021. In the forest plot, we fitted an approximate curve with 95% confidence intervals (CIs) for the mean prevalence of SHT exposure over the study period. In analysis 2, we constructed a scatter plot of urinary cotinine levels and a forest plot of the prevalence of SHT exposure according to the household tobacco use status. In addition, using *t*-tests, we assessed the differences in the mean prevalence of SHT exposure according to household tobacco use status.

In additional analyses, we assessed the annual trends from 2018 to 2021 in the prevalence of CC or HTP users among the children’s household members who were tobacco users. In addition, we assessed the number of tobacco users among household members by survey year, from 2011 to 2021. All analyses were conducted using Stata (version 17.0; Stata Corp., College Station, TX, USA). Two-sided *p*-values <.05 were considered statistically significant in all cases. This study followed the strengthening the reporting of observational studies in epidemiology (STROBE) guidelines.

### Ethical Considerations

This study was approved by the Ethics Committee of Tohoku University Graduate School of Dentistry (approval number: 34020). This study was based on the de-identifiable data approved to be used and provided by the Education Board of Kumagaya City. Informed consent was obtained from the children’s parents or guardians by the Education Board of Kumagaya City.

## Results

Table 1 presents the characteristics of the analytical sample. Among the 15 927 participants (male, 8250; female, 7677), 1887 (11.8%) were exposed to SHT as assessed by urinary cotinine levels. There were 7999 (50.2%) participants who had at least one tobacco user in their household. In addition, among the participants exposed to SHT, 1727 (91.5%) had at least one tobacco user in their household. Among the participants’ household members, the prevalence of tobacco users was 41.3% for fathers, 17.9% for mothers, 2.3% for grandparents, 0.4% for siblings, and 0.8% for others.

**Table 1.** Characteristics of Analytical Sample According to SHT Exposure Status

	Total (*n* = 15 927)	SHT exposure^*^	
		No (*n* = 14 040)	Yes (*n* = 1887)
	*n* (col. %)	*n* (col. %)	*n* (col. %)
**Sex**			
Male	8250 (51.8)	7272 (51.8)	978 (51.8)
Female	7677 (48.2)	6768 (48.2)	909 (48.2)
Having at least one tobacco user in household			
No	7928 (49.8)	7768 (55.3)	160 (8.5)
Yes	7999 (50.2)	6272 (44.7)	1727 (91.5)
Number of tobacco users in household			
0	7928 (49.8)	7768 (55.3)	160 (8.5)
1	6085 (38.2)	5134 (36.6)	951 (50.4)
2	1847 (11.6)	1105 (7.9)	742 (39.9)
≥3	67 (0.4)	33 (0.2)	34 (1.8)
Household member who was a tobacco user			
Father			
No^†^	9349 (58.7)	8728 (62.2)	621 (32.9)
Yes	6578 (41.3)	5312 (37.8)	1266 (67.1)
Mother			
No^†^	13 082 (82.1)	12 306 (87.6)	776 (41.1)
Yes	2845 (17.9)	1734 (12.4)	1111 (58.9)
Grandparents			
No^†^	15 561 (97.7)	13 796 (98.3)	1765 (93.5)
Yes	366 (2.3)	244 (1.7)	122 (6.5)
Siblings			
No^†^	15 864 (99.6)	13 998 (99.7)	1866 (98.9)
Yes	63 (0.4)	42 (0.3)	21 (1.1)
Others			
No^†^	15 797 (99.2)	13 928 (99.2)	1869 (99.0)
Yes	130 (0.8)	112 (0.8)	18 (1.0)

SHT = secondhand tobacco.

^*^The participants with urinary cotinine levels≥5.0 ng/mL were classified into the cotinine-assessed SHT-exposed group.

^†^If the household did not have a member, the participants were classified into “no” group because our questionnaire could not distinguish whether the household did not have a member or the member was a non-tobacco user (both were recorded in the same way).

**Figure 1.**
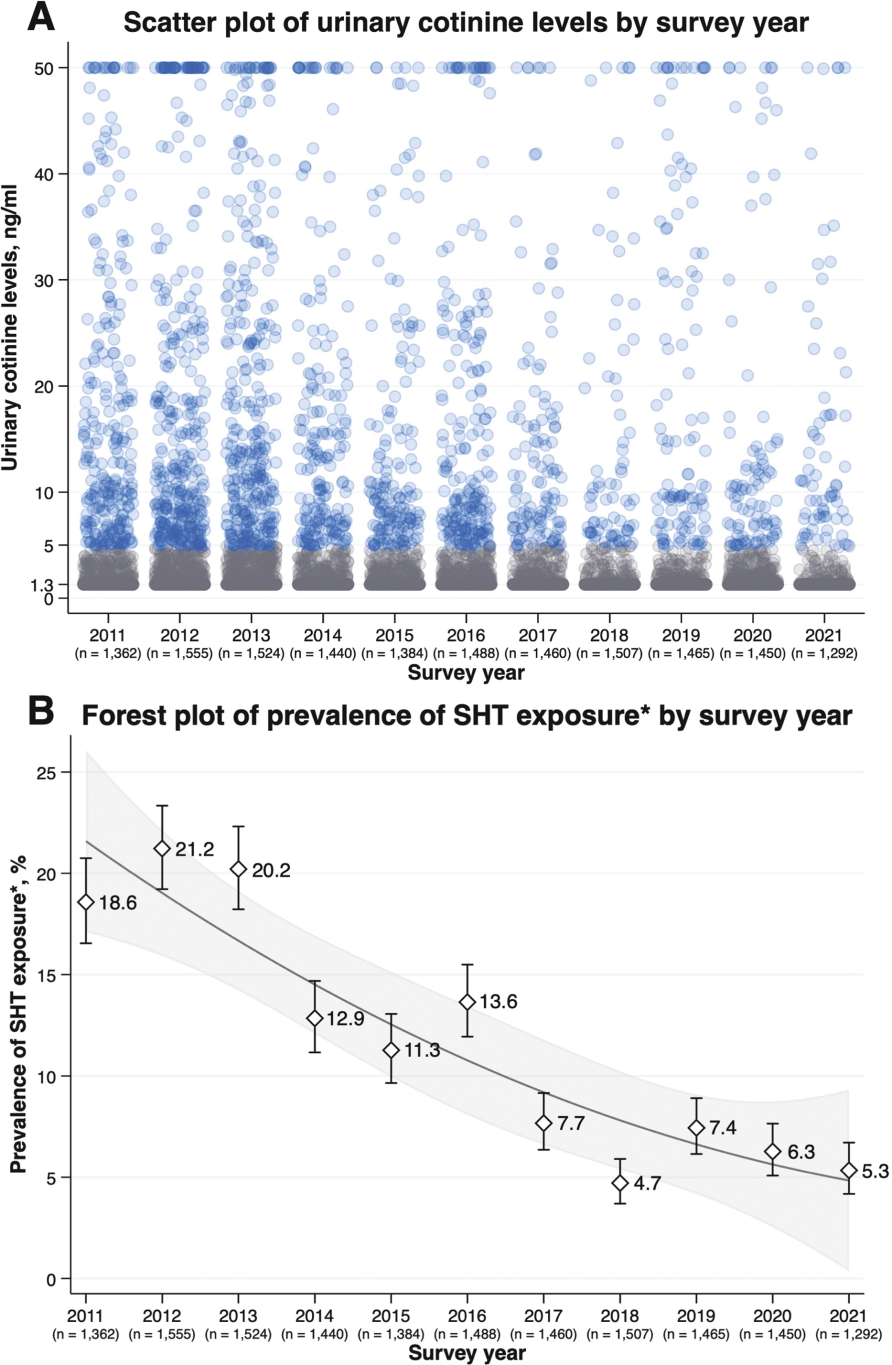
(A) Scatter plot of urinary cotinine level by survey year (B) forest plot of prevalence of SHT exposure by survey year. SHT = secondhand tobacco. The “*n*” below the survey year denotes the number of children included in the analysis. (A) A blue circle denotes a participant with urinary cotinine levels ≥5.0 ng/mL and a gray circle denotes that <5.0 ng/mL. The participants with urinary cotinine levels>50.0 ng/mL were truncated at 50.0 ng/mL (*n* = 156). (B) The participants with urinary cotinine levels ≥5.0 ng/mL were classified into the SHT-exposed group.

Figure 1 A is a scatter plot of urinary cotinine level by survey year that shows a declining trend in the number of participants with a urinary cotinine level ≥5.0 ng/mL from 2011 to 2021. In addition, there was a smaller number of participants with urinary cotinine levels≥10.0 ng/mL in later years. Figure 1 B is a forest plot of the prevalence of SHT exposure by survey year that shows a declining trend in the prevalence of SHT exposure from 2011 to 2021. The prevalence declined from approximately 20% in 2011–2013 to 5% in 2018–2021. A similar decreasing trend over the survey years was observed in the number of participants with urinary cotinine levels above the limit of quantitation (Table S2).

**Figure 2.**
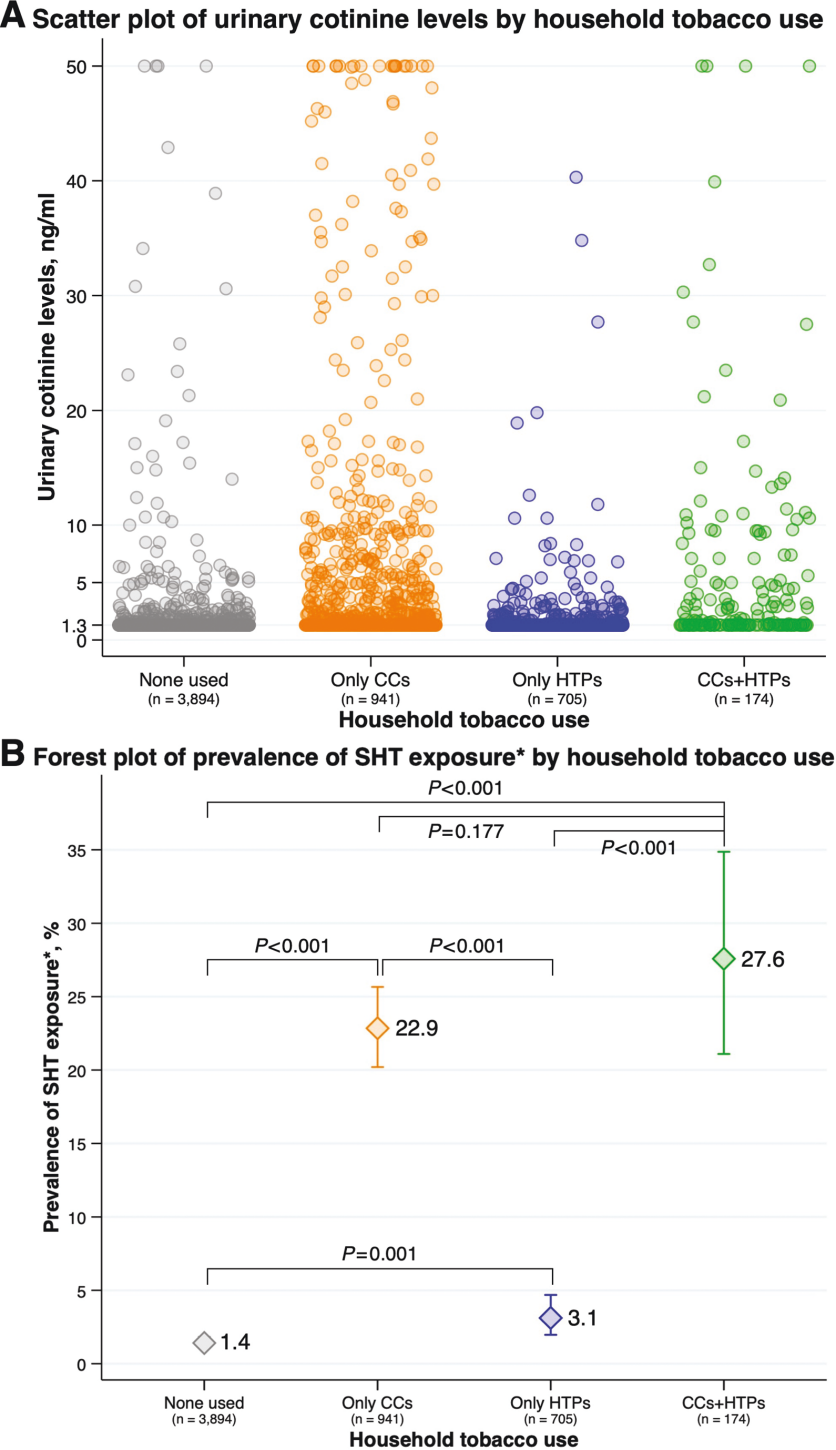
(A) Scatter plot of urinary cotinine level by household tobacco use status (B) forest plot of prevalence of SHT exposure by household tobacco use status. CCs = combustible cigarettes; HTPs = heated tobacco products; SHT = secondhand tobacco. The “*n*” below the survey year denotes the number of children included in the analysis. (A) A circle denotes a participant included in the analysis. The participants with urinary cotinine levels>50.0 ng/mL were truncated at 50.0 ng/mL (*n* = 27). (B) The participants with urinary cotinine levels ≥5.0 ng/mL were classified into the SHT-exposed group.

Figure 2A is a scatter plot of urinary cotinine level by household tobacco use status that shows 3894 (68.1%) participants did not have tobacco users, 941 (16.5%) had only CC users, 705 (12.3%) had only HTP users, and 174 (3.0%) had both CC users and HTP users in their household. There were several fractions of participants with a urinary cotinine level ≥5.0 ng/mL, particularly in the households that had CC users. Figure 2B is a forest plot of the prevalence of SHT exposure by household tobacco use status showing that compared with the participants who did not have tobacco users in their household, the prevalence of SHT exposure was higher in those who had only CC users (*p* < .001), only HTP users (*p* = .001), and both CC users and HTP users (*p* < .001). In addition, compared with the participants who had only HTP users in their household, the prevalence of SHT exposure was higher in those who had only CC users (*p* < .001) and both CC users and HTP users (*p* < .001). Conversely, there was no evidence of differences in the prevalence of SHT exposure between the participants who had only CC users and both CC users and HTP users (*p* = .177). In addition, similar patterns of SHT exposure status according to household tobacco use status were observed across the survey year (Table S3).

Similar results were observed in the sensitivity analysis that used a different cutoff (urinary cotinine level ≥3.0 ng/mL) in the definition of SHT exposure. The prevalence of SHT exposure showed a declining trend from 2011 to 2021, and the prevalence declined from approximately 25% in 2011–2013 to 10% in 2018–2021 (Figure S1). In addition, the prevalence of SHT exposure was higher in the participants with CC or HTP users in their households than in those who did not have tobacco users. Furthermore, the prevalence of SHT exposure was higher in participants with CC users in their households than in those with HTP users (Figure S2). In the additional analysis, there was an increasing trend in the prevalence of HTP users among tobacco users in the household, whereas the prevalence of CC users showed a declining trend (Figure S3). In addition, there was a decreasing trend in the prevalence of tobacco users, with the prevalence of tobacco users declining from 46.5% in 2011 to 35.8% in 2021 in fathers, and from 19.2% in 2011 to 12.6% in 2021 in mothers (Table S4).

## Discussion

In this 11-year repeated cross-sectional study of 15 927 elementary school children, we observed that the prevalence of SHT exposure, assessed by urinary cotinine levels, among children in Japan has decreased from approximately 20% in the early 2010s to approximately 5% in the early 2020s. However, there were evident differences in urinary cotinine levels according to their household tobacco use status. Compared to children without tobacco users in the household, the prevalence of SHT exposure was higher among those with tobacco users, regardless of the type of tobacco product. This result suggests that children were exposed to SHT even in households where only HTPs were used, and some actions would be required to protect children from the potential harms of SHT exposure.

A previous study^27^ observed a decrease in SHT exposure among people in middle adolescence in Japan during a similar period (2008–2017) as our study; however, the current situation of SHT exposure among the younger population remained unknown. Our study found a continuous decrease in SHT exposure among people in early adolescence from 2011 to 2021, extending our understanding of the current situation of SHT exposure among children in Japan. Similarly, a recent study which used cotinine data found that there was a decreasing trend of secondhand smoke exposure among children from 1998 to 2018 in England.^33^ Considering that the number of tobacco users has been decreasing in Japan (as supported by the results in Table S2), the decrease in SHT exposure among children may be explained by a decrease in the likelihood that tobacco users would be in the presence of children. In addition, the Revised Health Promotion Act prohibited tobacco use in public places from April 2020, in Japan^34^; hence, SHT exposure is expected to decrease further in the coming years. However, when a similar act prohibiting tobacco use in indoor public places was implemented in Spain, it did not reduce SHT exposure in children.^35^ Therefore, the prevalence of SHT exposure among children should be continuously monitored to evaluate the effectiveness of the Revised Health Promotion Act and provide feedback to refine the act.

This study revealed that the prevalence of SHT exposure was lower among children with only HTP users in their household than those with CC users. This may reflect that HTPs contain the same or lower amounts of nicotine as CCs per stick.^36^ However, HTPs have been reported to contain more harmful and potentially harmful constituents,^37^ including some with unknown long-term effects on health. In this context, it is important to give more attention to evaluating SHT exposure using a biomarker to determine whether it adequately captures the exposure. Our results in Figures S1 and S2 encourage the use of a stricter cutoff for urinary cotinine levels (≥3.0 ng/mL) in the definition of SHT exposure to account for the use of a variety of tobacco products.

The strength of this study includes that this is the first large-scale examination of trends in SHT exposure, assessed by urinary cotinine levels, among Japanese elementary school students over a 10-year period. In addition, this study obtained information on the household use of tobacco products, including HTPs, which is an emerging public health problem. However, this study has several limitations. First, this study was based on data from elementary school students in a municipality. Therefore, our findings should be interpreted with caution, as they may not be generalizable to the same age group not attending school or to elementary school students in other municipalities. However, the latest survey by the Ministry of Education, Culture, Sports, Science, and Technology (MEXT) reported that the percentage of students not attending elementary schools is 1.7% in Japan.^38^ Second, although this study focused on the presence of tobacco users in households as one of the causes of SHT exposure, the possibility that children were exposed to SHT outside the home (eg, on school roads) cannot be ruled out. However, since our study subjects were 10-year-old elementary school children who generally spend most of their time at home,^39^ we believe that SHT exposure outside the home is unlikely to greatly affect our findings. Third, the limit of quantitation for urinary cotinine levels was relatively higher in our study (1.3 ng/mL) than in other studies.^40^ Therefore, it remains possible that we did not capture individuals who were actually exposed to SHT but whose urinary cotinine levels were lower than the limit of quantitation. However, since we defined SHT exposure using a cutoff value sufficiently high from the limit of quantitation (≥5.0 ng/mL in the main analysis and ≥3.0 ng/mL in the sensitivity analysis), this would not greatly affect our findings.

In conclusion, this study found a consistent decreasing trend in the prevalence of SHT exposure from 2011 to 2021 among school-aged children in Japan. In addition, the prevalence of SHT exposure was higher among children in households with tobacco users, regardless of the type of tobacco product, than among those in households without tobacco users. Our findings suggest a possibility that the use of tobacco products, including HTPs, in households with children should be considered for legal regulation to protect people who cannot choose whether to accept the risks associated with SHT exposure.

## Supplementary material

Supplementary material is available at *Nicotine and Tobacco Research* online.

ntae220_suppl_Supplementary_Tables_S1-S4_Figures_S1-S3

## Data Availability

All data used in this study are not publicly available due to ethical or legal restrictions.
