# Supplementary material for: Secondhand Tobacco Exposure Assessed Using Urinary Cotinine Among 10-Year-Old Children in Japan: An 11-Year Repeated Cross-sectional Study
Source: Nicotine Tob Res. 2024 Sep 19;27(3):534–41. doi: 10.1093/ntr/ntae220 (PMC11847774; doi:10.1093/ntr/ntae220)
Supplement: ntae220_suppl_Supplementary_Tables_S1-S4_Figures_S1-S3 [file ntae220_suppl_supplementary_tables_s1-s4_figures_s1-s3.docx]

Supplementary material (online only)

**Title:**

Secondhand tobacco exposure assessed using urinary cotinine among 10-year-old children in Japan: An 11-year repeated cross-sectional study

**Authors:**

Yudai Tamada, Kenji Takeuchi, Takahiro Tabuchi

**Table of Contents**

[**Supplemental Table 1.** Number of participants who did not have urinary cotinine information by survey year (2011–2021). 2](#_Toc171273721)

[**Supplemental Table 2.** Number of participants with urinary cotinine levels above limit of quantitation by survey year (2011–2021). 3](#_Toc171273722)

[**Supplemental Table 3.** SHT exposure status according to household tobacco use status by survey year (2018–2021). 4](#_Toc171273723)

[**Supplemental Table 4.** Number of tobacco product users among household members by survey year (2011–2021). 5](#_Toc171273724)

[**Supplemental Figure 1.** Forest plot of prevalence of SHT exposure by survey year using a different cut-off (≥3.0 ng/ml) in definition of SHT exposure. 6](#_Toc171273726)

[**Supplemental Figure 2.** Forest plot of prevalence of SHT exposure by household tobacco use using a different cut-off (≥3.0 ng/ml) in definition of SHT exposure. 7](#_Toc171273727)

[**Supplemental Figure 3.** Annual trends from 2018 to 2021 on prevalence of CCs or HTPs users among tobacco users in household. 8](#_Toc171273728)

# **Supplemental Table 1.** Number of participants who did not have urinary cotinine information by survey year (2011–2021).

| **Survey year** | **Having urinary cotinine information**,  n (row %) | **Not having urinary cotinine information**,  n (row %) | **Total** |
| --- | --- | --- | --- |
| 2011 | 1,362 (99.3) | 9 (0.7) | 1,371 (100.0) |
| 2012 | 1,555 (87.3) | 227 (12.7) | 1,782 (100.0) |
| 2013 | 1,524 (89.3) | 183 (10.7) | 1,707 (100.0) |
| 2014 | 1,440 (89.1) | 176 (10.9) | 1,616 (100.0) |
| 2015 | 1,384 (87.9) | 190 (12.1) | 1,574 (100.0) |
| 2016 | 1,488 (90.2) | 161 (9.8) | 1,649 (100.0) |
| 2017 | 1,460 (87.2) | 215 (12.8) | 1,675 (100.0) |
| 2018 | 1,507 (93.8) | 100 (6.2) | 1,607 (100.0) |
| 2019 | 1,465 (93.0) | 111 (7.0) | 1,576 (100.0) |
| 2020 | 1,450 (91.2) | 140 (8.8) | 1,590 (100.0) |
| 2021 | 1,292 (89.2) | 157 (10.8) | 1,449 (100.0) |
| Total | 15,927 (90.5) | 1,669 (9.5) | 17,596 (100.0) |

# **Supplemental Table 2.** Number of participants with urinary cotinine levels above limit of quantitation by survey year (2011–2021).

| **Survey year** | **Under limit of quantitation**,  n (row %) | **Above limit of quantitation**,  n (row %) | **Total** |
| --- | --- | --- | --- |
| 2011 | 905 (66.5) | 457 (33.6) | 1,362 (100.0) |
| 2012 | 964 (62.0) | 591 (38.0) | 1,555 (100.0) |
| 2013 | 930 (61.0) | 594 (39.0) | 1,524 (100.0) |
| 2014 | 1,073 (74.5) | 367 (25.5) | 1,440 (100.0) |
| 2015 | 1,050 (75.9) | 334 (24.1) | 1,384 (100.0) |
| 2016 | 1,073 (72.1) | 415 (27.9) | 1,488 (100.0) |
| 2017 | 1,189 (81.4) | 271 (18.6) | 1,460 (100.0) |
| 2018 | 1,334 (88.5) | 173 (11.5) | 1,507 (100.0) |
| 2019 | 1,238 (84.5) | 227 (15.5) | 1,465 (100.0) |
| 2020 | 1,139 (78.6) | 311 (21.5) | 1,450 (100.0) |
| 2021 | 1,142 (88.4) | 150 (11.6) | 1,292 (100.0) |
| Total | 12,037 (75.6) | 3,890 (24.4) | 15,927 (100.0) |

Notes: The limit of quantitation for urinary cotinine levels was 1.3 ng/ml in our methods.

# **Supplemental Table 3.** SHT exposure status according to household tobacco use status by survey year (2018–2021).

|  | Survey year | | | | Total, n (col %) |
| --- | --- | --- | --- | --- | --- |
| SHT exposure* | 2018, n (col %) | 2019, n (col %) | 2020, n (col %) | 2021, n (col %) |  |
| Household tobacco use |  |  |  |  |  |
| None used |  |  |  |  |  |
| No | 1,002 (98.5) | 968 (98.4) | 983 (98.7) | 886 (98.8) | 3,839 (98.6) |
| Yes | 15 (1.5) | 16 (1.6) | 13 (1.3) | 11 (1.2) | 55 (1.4) |
| Only CCs |  |  |  |  |  |
| No | 221 (83.7) | 205 (73.5) | 160 (73.7) | 140 (77.4) | 726 (77.2) |
| Yes | 43 (16.3) | 74 (26.5) | 57 (26.3) | 41 (22.7) | 215 (22.9) |
| Only HTPs |  |  |  |  |  |
| No | 173 (97.7) | 156 (96.9) | 192 (95.1) | 162 (98.2) | 683 (96.9) |
| Yes | 4 (2.3) | 5 (3.1) | 10 (5.0) | 3 (1.8) | 22 (3.1) |
| CCs + HTPs |  |  |  |  |  |
| No | 40 (81.6) | 27 (65.9) | 24 (68.6) | 35 (71.4) | 126 (72.4) |
| Yes | 9 (18.4) | 14 (34.2) | 11 (31.4) | 14 (28.6) | 48 (27.6) |

Abbreviations: SHT = secondhand tobacco; CCs = combustible cigarettes; HTPs = heated tobacco products.

Notes: This analysis used data from 2018 to 2021 because the question regarding HTP use status was included in the questionnaire from 2018.

* The participants with urinary cotinine level ≥5.0 ng/ml were classified into the cotinine assessed SHT exposed group.

# **Supplemental Table 4.** Number of tobacco product users among household members by survey year (2011–2021).

|  | **Household member** | | | | | | | | | | **Total** |
| --- | --- | --- | --- | --- | --- | --- | --- | --- | --- | --- | --- |
|  | **Father** | | **Mother** | | **Grandparents** | | **Siblings** | | **Others** | |  |
| **Survey**  **year** | **Non user**,  n (row %) | **User**,  n (row %) | **Non user**,  n (row %) | **User**,  n (row %) | **Non user**,  n (row %) | **User**,  n (row %) | **Non user**,  n (row %) | **User**,  n (row %) | **Non user**,  n (row %) | **User**,  n (row %) |  |
| 2011 | 729 (53.5) | 633 (46.5) | 1,100 (80.8) | 262 (19.2) | 1,323 (97.1) | 39 (2.9) | 1,362 (100.0) | 0 (0.0) | 1,359 (99.8) | 3 (0.2) | 1,362 (100.0) |
| 2012 | 876 (56.3) | 679 (43.7) | 1,255 (80.7) | 300 (19.3) | 1,537 (98.8) | 18 (1.2) | 1,550 (99.7) | 5 (0.3) | 1,545 (99.4) | 10 (0.6) | 1,555 (100.0) |
| 2013 | 834 (54.7) | 690 (45.3) | 1,248 (81.9) | 276 (18.1) | 1,489 (97.7) | 35 (2.3) | 1,523 (99.9) | 1 (0.1) | 1,519 (99.7) | 5 (0.3) | 1,524 (100.0) |
| 2014 | 785 (54.5) | 655 (45.5) | 1,186 (82.4) | 254 (17.6) | 1,348 (93.6) | 92 (6.4) | 1,436 (99.7) | 4 (0.3) | 1,415 (98.3) | 25 (1.7) | 1,440 (100.0) |
| 2015 | 794 (57.4) | 590 (42.6) | 1,020 (73.7) | 364 (26.3) | 1,384 (100.0) | 0 (0.0) | 1,378 (99.6) | 6 (0.4) | 1,370 (99.0) | 14 (1.0) | 1,384 (100.0) |
| 2016 | 863 (58.0) | 625 (42.0) | 1,132 (76.1) | 356 (23.9) | 1,488 (100.0) | 0 (0.0) | 1,478 (99.3) | 10 (0.7) | 1,473 (99.0) | 15 (1.0) | 1,488 (100.0) |
| 2017 | 884 (60.6) | 576 (39.5) | 1,159 (79.4) | 301 (20.6) | 1,460 (100.0) | 0 (0.0) | 1,448 (99.2) | 12 (0.8) | 1,442 (98.8) | 18 (1.2) | 1,460 (100.0) |
| 2018 | 937 (62.2) | 570 (37.8) | 1,331 (88.3) | 176 (11.7) | 1,452 (96.4) | 55 (3.7) | 1,501 (99.6) | 6 (0.4) | 1,497 (99.3) | 10 (0.7) | 1,507 (100.0) |
| 2019 | 895 (61.1) | 570 (38.9) | 1,252 (85.5) | 213 (14.5) | 1,421 (97.0) | 44 (3.0) | 1,461 (99.7) | 4 (0.3) | 1,452 (99.1) | 13 (0.9) | 1,465 (100.0) |
| 2020 | 922 (63.6) | 528 (36.4) | 1,270 (87.6) | 180 (12.4) | 1,409 (97.2) | 41 (2.8) | 1,442 (99.5) | 8 (0.6) | 1,442 (99.5) | 8 (0.6) | 1,450 (100.0) |
| 2021 | 830 (64.2) | 462 (35.8) | 1,129 (87.4) | 163 (12.6) | 1,250 (96.8) | 42 (3.3) | 1,285 (99.5) | 7 (0.5) | 1,283 (99.3) | 9 (0.7) | 1,292 (100.0) |
| Total | 9,349 (58.7) | 6,578 (41.3) | 13,082 (82.1) | 2,845 (17.9) | 15,561 (97.7) | 366 (2.3) | 15,864 (99.6) | 63 (0.4) | 15,797 (99.2) | 130 (0.8) | 15,927 (100.0) |

# Notes: If the household did not have the member, the participants were classified into “non user” group because our questionnaire could not distinguish whether the household did not have the member or the member was a non tobacco user (both were recorded in the same way).

# **Supplemental Figure 1.** Forest plot of prevalence of SHT exposure by survey year using a different cut-off (≥3.0 ng/ml) in definition of SHT exposure.


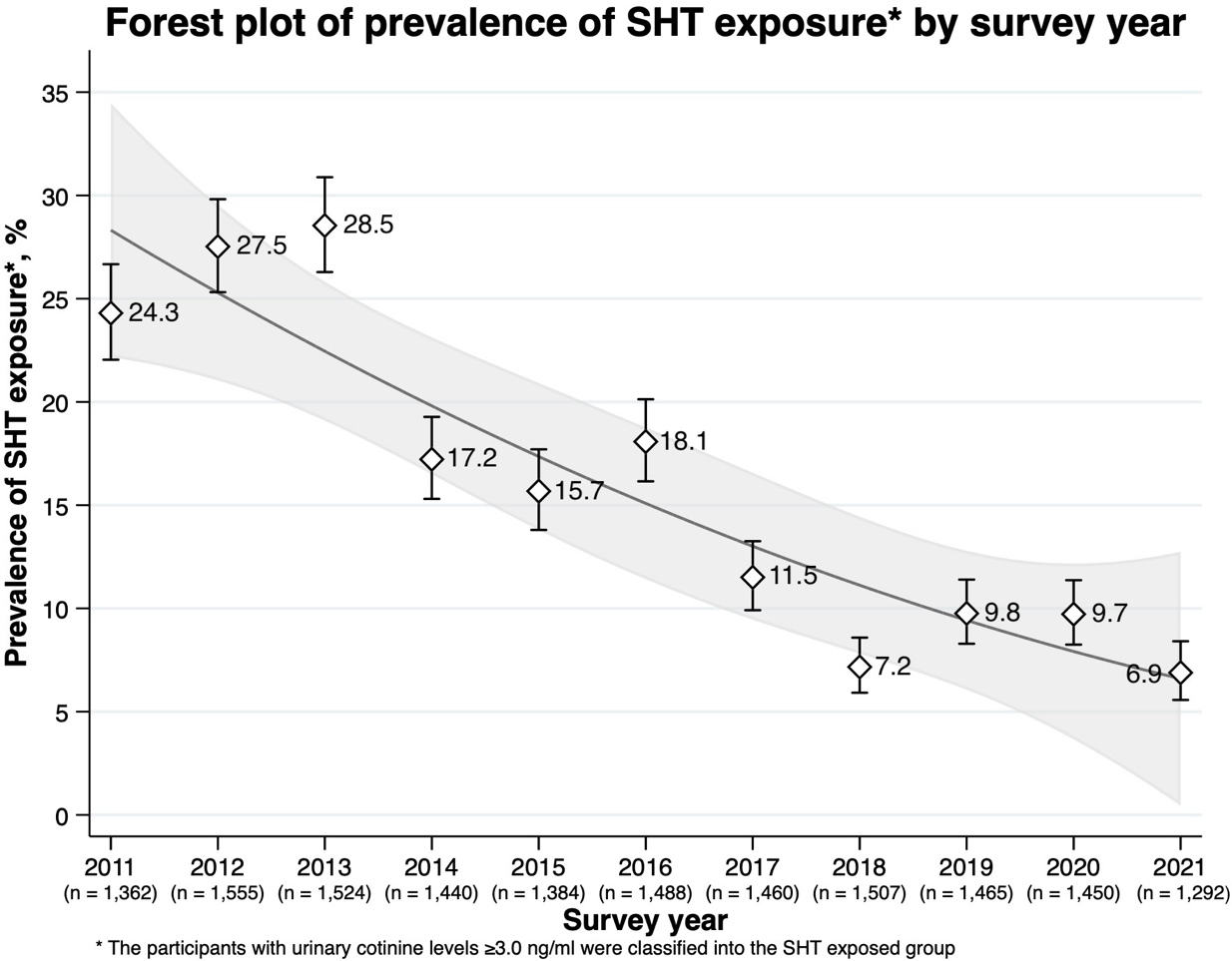


Abbreviations: SHT = secondhand tobacco.

Alt text: Graph of statistical data that shows “forest plot displaying the trend of decreasing prevalence of SHT exposure from 2011 to 2021 using a different cut-off (≥3.0 ng/ml) in the definition of SHT exposure.”

# **Supplemental Figure 2.** Forest plot of prevalence of SHT exposure by household tobacco use using a different cut-off (≥3.0 ng/ml) in definition of SHT exposure.


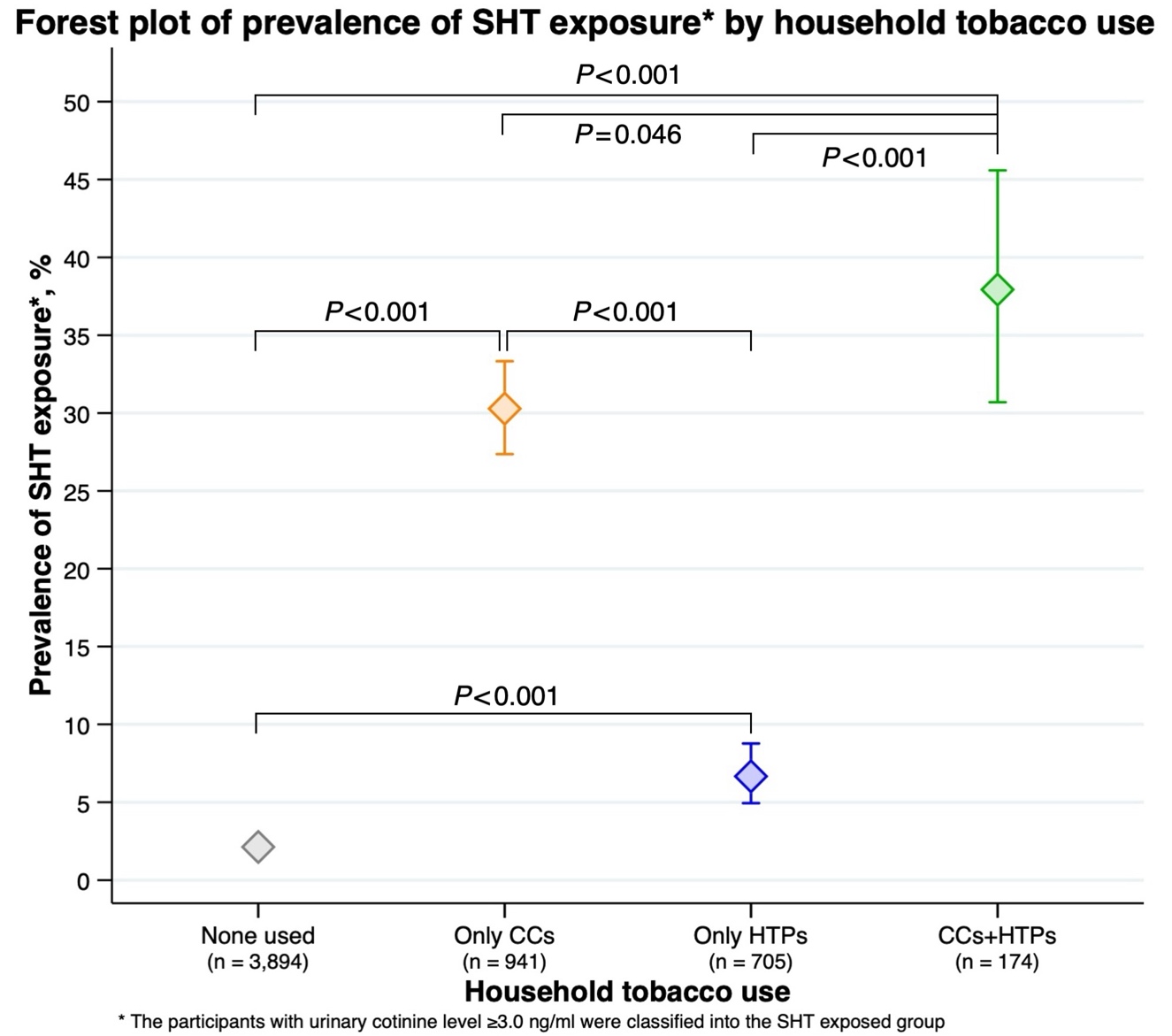


Abbreviations: CCs = combustible cigarettes; HTPs = heated tobacco products; SHT = secondhand tobacco.

Alt text: Graph of statistical data that shows “forest plot displaying the differences of prevalence of SHT exposure by household tobacco use status (none used/only CCs/only HTPs/CCs+HTPs) using a different cut-off (≥3.0 ng/ml) in the definition of SHT exposure.”

# **Supplemental Figure 3.** Annual trends from 2018 to 2021 on prevalence of CCs or HTPs users among tobacco users in household.


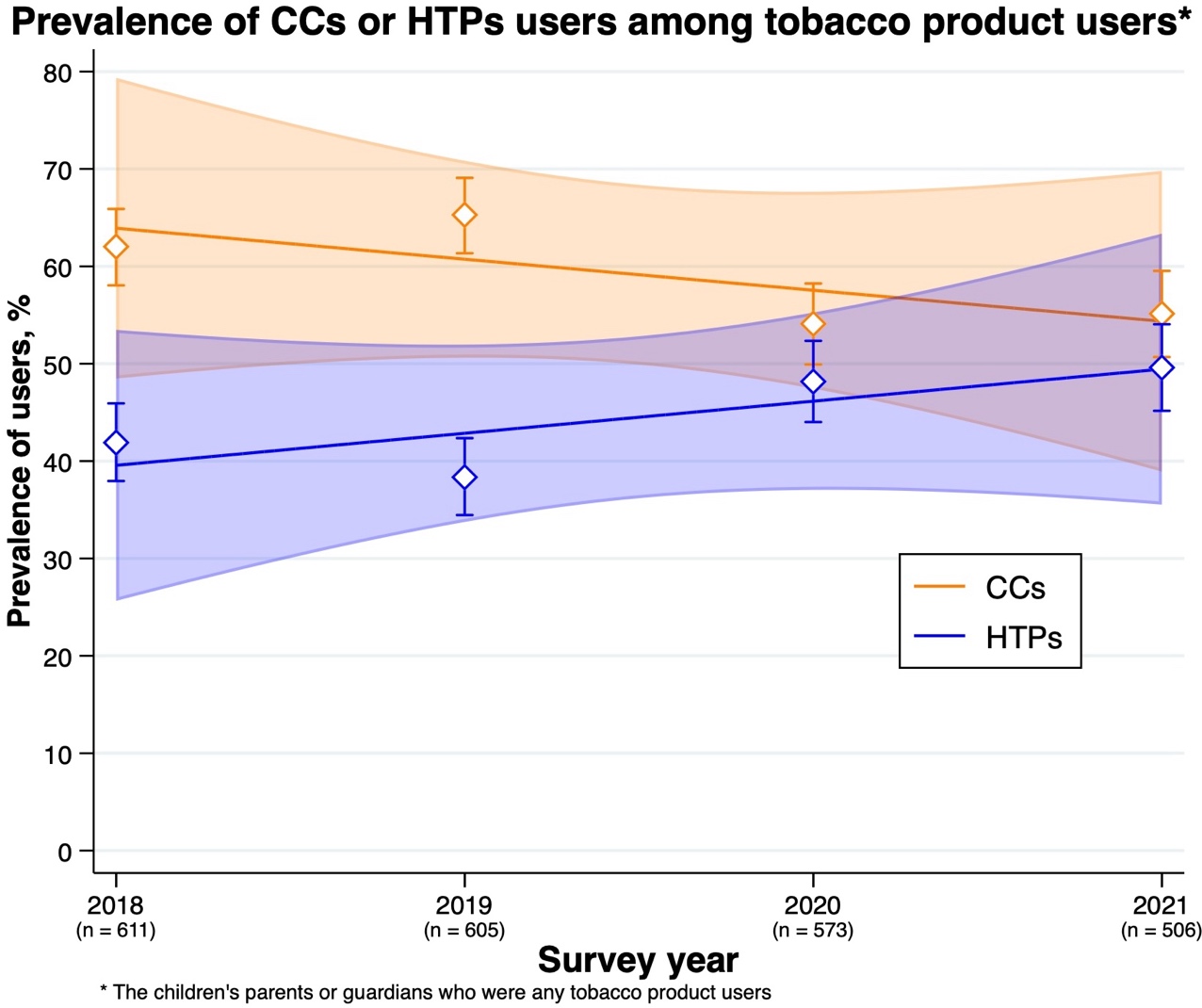


Abbreviations: CCs = combustible cigarettes; HTPs = heated tobacco products.

Alt text: Graph of statistical data that shows “forest plot displaying the trend of decreasing prevalence of CCs users and increasing prevalence of HTPs users among tobacco users in household from 2018 to 2021.”
